# A One Health Approach Molecular Analysis of *Staphylococcus aureus* Reveals Distinct Lineages in Isolates from Miranda Donkeys (*Equus asinus*) and Their Handlers

**DOI:** 10.3390/antibiotics11030374

**Published:** 2022-03-10

**Authors:** Vanessa Silva, Cláudia Alfarela, Manuela Caniça, Vera Manageiro, Miguel Nóvoa, Belen Leiva, Maria Kress, José Luís Capelo, Patrícia Poeta, Gilberto Igrejas

**Affiliations:** 1 Microbiology and Antibiotic Resistance Team (MicroART), Department of Veterinary Sciences, University of Trás-os-Montes and Alto Douro (UTAD), 5000-801 Vila Real, Portugal; vanessasilva@utad.pt (V.S.); claudiavpa@hotmail.com (C.A.); 2 Department of Genetics and Biotechnology, University of Trás-os-Montes and Alto Douro (UTAD), 5000-801 Vila Real, Portugal; gigrejas@utad.pt; 3 Functional Genomics and Proteomics Unit, University of Trás-os-Montes and Alto Douro (UTAD), 5000-801 Vila Real, Portugal; 4 Associated Laboratory for Green Chemistry (LAQV@REQUIMTE), University NOVA of Lisboa, 2825-466 Lisbon, Portugal; 5 National Reference Laboratory of Antibiotic Resistances and Healthcare Associated Infections (NRL-AMR/HAI), Department of Infectious Diseases, National Institute of Health Dr Ricardo Jorge, Av. Padre Cruz, 1649-016 Lisbon, Portugal; manuela.canica@insa.min-saude.pt (M.C.); vera.manageiro@insa.min-saude.pt (V.M.); 6 Centre for the Studies of Animal Science, Institute of Agrarian and Agri-Food Sciences and Technologies, Oporto University, 4051-401 Oporto, Portugal; 7 AEPGA, Association for the Study and Protection of Asinines, Atenor, 5225-011 Miranda do Douro, Portugal; miguelnovoa@aepga.pt (M.N.); belenleiva.aepga@gmail.com (B.L.); mariakress.aepga@gmail.com (M.K.); 8 BIOSCOPE Group, LAQV@REQUIMTE, Chemistry Department, Faculty of Science and Technology, NOVA University of Lisbon, 2825-466 Almada, Portugal; jlcm@fct.unl.pt; 9 Proteomass Scientific Society, 2825-466 Almada, Portugal; 10 CECAV—Veterinary and Animal Research Centre, University of Trás-os-Montes and Alto Douro (UTAD), 5000-801 Vila Real, Portugal; 11 Associate Laboratory for Animal and Veterinary Science (AL4AnimalS), University of Trás-os-Montes and Alto Douro (UTAD), 5000-801 Vila Real, Portugal

**Keywords:** *Staphylococcus aureus*, CoNS, donkeys, animal-to-human transmission

## Abstract

Donkeys (*Equus asinus*) are in decline in Europe. Occupational exposure to farm animals has been associated with increased staphylococci carriage. We aimed to isolate *S. aureus* and coagulase-negative staphylococci (CoNS) from donkeys and handlers and characterize the antimicrobial resistance profiles and genetic lineages of *S. aureus* strains. Oral and nasal swab samples were collected from 49 Miranda donkeys and 23 handlers from 15 different farms. Staphylococci species were identified by MALDI-TOF MS. The presence of antimicrobial resistance genes and virulence factors was investigated by PCR. Molecular typing was performed in *S. aureus* isolates. From the 49 donkey samples, 4 *S. aureus* (8.2%) and 21 CoNS (42.9%) were isolated. Ten handlers (43.5%) were carriers of *S. aureus* and 4 (17.4%) carried CoNS. The CoNS isolates showed resistance to several classes of antimicrobials encoded by the *mec*A, *aph* (3′)-IIIa, *ant* (4′)-Ia, *tet*M, *tet*K, *lnu*A, *erm*B, *erm*C, *dfr*A and *dfr*G genes. *S. aureus* isolates were resistant to penicillin, aminoglicosides and tetracycline harboring the *bla*Z, *aph* (3′)-IIIa, *tet*L, *tet*M and *tet*K genes. All *S. aureus* isolates from donkeys belonged to ST49 and *spa*-type t208 while the strains isolated from the handlers were ascribed to 3 STs and 7 *spa*-types. However, human isolates were from different STs than the donkey isolates. Donkeys are mainly colonized by methicillin-resistant *S. sciuri*. *S. aureus* transmission between donkeys and their handlers appears not to have occurred since the isolates belonged to different genetic lineages.

## 1. Introduction

*Staphylococcus* spp. are widely disseminated worldwide and have been isolated from human infections, community humans, pets, livestock, wild animals and the natural environment [1]. Both *Staphylococcus aureus* and coagulase-negative staphylococci (CoNS) are commensal bacteria that colonize the skin and mucosa of humans and animals [2]. Nevertheless, staphylococci are opportunist pathogens that are responsible for a wide-range of infections, including skin and soft-tissue infection, septicemia and endocarditis [3]. Staphylococci, particularly *S. aureus*, have the ability to easily acquire antimicrobial resistance determinants [4]. Methicillin resistance in staphylococci is conferred by the acquisition of the *mec* genes (*mec*A, *mec*B or *mec*C), which are carried by the staphylococcal cassette chromosome *mec* (SCC*mec*) [5]. Methicillin-resistant *S. aureus* (MRSA) are usually associated with complicated infections representing a public health concern [6]. It is considered that the *mec*A gene may have originated from some species of CoNS since homologues of this gene have been found in the *S. sciuri* group species [4]. CoNS carrying the *mec*A gene may act as potential donors leading to the emergence of methicillin-resistant *S. aureus* (MRSA) clones [7].

Animals can be a source of *staphylococci* zoonotic infections, particularly the clones that possess no host specificity [2]. It has also been demonstrated that the close contact between animals and humans can facilitate the transmission of pathogens. Staphylococci transmission events have been documented mainly between pets and owners and livestock and their handlers [8,9]. Molecular typing techniques, such as multilocus sequence typing (MLST), have provided information on the evolution of human and animal strains and shown that humans were the main hosts of *S. aureus*, but due to the occurrence of transmission it had acquired the ability to infect animals [10]. The frequent human-to-animal transmission eventually led to specific strain lineages adapting to new animal hosts [10,11]. MLST analysis showed that some sequence types (STs) and clonal complexes (CCs) are predominant in animal *S. aureus*, such as CC398, CC9, CC1, CC97, CC133 and CC121, and others are prevalent in human *S. aureus* (CC1, CC5, CC8, CC12, CC15, CC22, CC25, CC30, CC45 and CC51) [10]. The emergence of *S. aureus* and CoNS in equines, including horses, donkeys and mules, has been demonstrated [12,13,14]. The frequency of *S. aureus* colonization in horses ranges from 4% to 39% in Europe and CC8, CC22 or CC398 are the most common lineages identified [12,15,16]. Nevertheless, although studies reporting staphylococci in horses are common, studies in healthy donkeys are still very scarce [13].

The donkey population has been decreasing since 1970 but lately this trend has reversed, and it is estimated that the donkey population is 4.3 million globally [17]. In Europe, donkeys are mainly found in Portugal, Spain, Italy and Greece. Miranda donkey is a native Portuguese donkey breed that originated from the Trás-os-Montes region [18]. Donkeys played an important role in maintaining rural communities and sustainable farming practices. However, more recently, these animals began to be used as companion animals and in therapeutic activities and ecotourism [11]. Therefore, we aimed to study the prevalence of *S. aureus* and CoNS in healthy donkeys and their handlers as well as the antimicrobial resistance and genetic lineages of the isolates in order to investigate a possible human-to-animal transmission or vice-versa.

## 2. Results

### 2.1. Characterization of S. aureus Isolates

In this study, 49 and 23 swab samples were collected from Miranda donkeys and handlers, respectively. Staphylococci were recovered from all 15 farms sampled. *S. aureus* were isolated from 10 farms while CoNS were isolated from 8 farms. From the 49 donkey samples, 4 *S. aureus* were identified while 10 *S. aureus* were isolated from handlers’ samples. However, no MRSA has been identified. Three donkeys colonized by *S. aureus* were from the same farm and their ages ranged from 3 to 8 years (Supplementary Materials Table S1). However, none of these donkeys’ handlers were *S. aureus* carriers. All *S. aureus* from donkeys belonging to the same farm (isolates VS3111, VS3113 and VS3114) had resistance to penicillin and kanamycin conferred by the *bla*Z and *aph* (3′)-IIIa genes (Table 1). Furthermore, all 3 isolates were ascribed to ST49, *spa*-type t208 and *agr* type II. The *S. aureus* isolated from the remaining donkey showed resistance to aminoglycosides and tetracycline and carried the *aph* (3′)-IIIa and *tet*K genes. Yet, these isolates also belonged to ST49, t208 and *agr* II. Regarding the virulence genes, all four isolates harbored the *hla*, *hlb* and *hld* genes with the exception of isolate VS3111, which also carried the *tst* gene.

The 10 *S. aureus* isolated from handlers had similar resistance patterns, showing resistance to penicillin, aminoglycosides, tetracycline and fusidic acid. The isolates carried the *bla*Z, *aph* (3′)-IIIa, *tet*L and *tet*M genes. *S. aureus* isolated from handlers carried the *hla* (*n* = 10), *hlb* (*n* = 7), *hld* (*n* = 10), *eta* (*n* = 3) and *tst* (*n* = 4) virulence genes. Isolates VS3101 and VS3102 also carried the *scn* and *sak* genes of the immune evasion cluster (IEC) system and were ascribed to type E. Both isolates were ascribed to ST1290 (CC1), *spa*-type t131 and *agr* I. Moreover, these isolates belonged to two handlers working at the same farm. Six isolates were ascribed to ST30 (CC30) and *agr* III. However, these isolates were distributed over four different *spa*-types. Finally, two isolates belonged to ST398 (CC398) and *spa*-types t571 and t011.

### 2.2. Characterization of CoNS Isolates

Donkey and handler samples were also screened for the presence of methicillin-resistant CoNS (MRCoNS). Only four (17.4%) handlers carried MRCoNS, these being 3 *S. epidermidis* and one *S. sciuri* (Table 2). Out of the 49 donkeys, 21 (42.9%) were colonized by CoNS including *S. sciuri* (*n* = 17), *S. lentus* (*n* = 2), *S. xylosus* and *S. vitulinus*. Two *S. epidermidis* were multidrug-resistant since they were resistant to four classes of antimicrobials. They were resistant to penicillin, aminoglycosides, erythromycin, tetracycline and trimethoprim/sulfamethoxazole and harbored the *bla*Z, *aac* (6′)-Ie-*aph* (2″)-Ia, *aph* (3′)-IIIa, *erm*C, *dfr*A and *dfr*G genes. The only *S. sciuri* isolated from one handler showed resistance penicillin, cefoxitin and clindamycin and harbored the *mec*A and *lnu*A genes. Even though all CoNS were isolated from ORSAB medium supplemented with 2.5 mg/L of oxacillin, two *S. epidermidis*, one *S. lentus* and one *S. xylosus* isolates lacked the *mec*A gene. All the remaining isolates caried the *mec*A gene even those showing phenotypic susceptibility to all antimicrobials tested (*n* = 7). One *S. lentus* isolate also displayed a multidrug-resistant phenotype with resistances to penicillin, ciprofloxacin, macrolides and licosamides conferred by the *mec*A, *bla*Z and *lnu*A genes. *S. sciuri* isolates showed a variety of resistances encoded by *optr*A, *aph* (3′)-IIIa, *ant* (4′)-Ia, *str*, *erm*B, *tet*K and *tet*M. Finally, only four CoNS carried virulence genes: two *eta*, one *tst* and one *hla*.

## 3. Discussion

In this study we collected oral and nasal swab samples from 49 Miranda donkeys and 23 handlers to investigate the frequency of staphylococci in donkeys and a possible transmission between animals and humans due to the close proximity. It has been shown that the close contact between pets and humans offers favorable conditions for transmission by direct contact [19]. In our study, *S. aureus* were isolated from 4 (8.2%) donkeys and 10 (43.5%) handlers among the 49 and 23 donkeys and handlers’ samples, respectively, and were further characterized by molecular typing in order to investigate a possible transmission. Studies reporting the infection or colonization of donkeys by *S. aureus* are very scarce. Nevertheless, one study conducted with nasal samples of healthy donkeys and another with conjunctival swabs reported a frequency of *S. aureus* of 50% and 47.8%, respectively [13,20]. Another study investigated the occurrence of *S. aureus* in healthy donkeys and donkeys with respiratory tract disease and reported an incidence of 13.2% [21]. As for handlers, the proportion of samples that were positive for *S. aureus* is in line with the normal frequency of human colonization, which is approximately 30% [22]. *S. aureus* from donkeys were ascribed to the same clonal lineage while *S. aureus* isolates from handlers belonged to several different lineages. All *S. aureus* isolated from donkeys belonged to ST49, *spa*-type t208 and *agr* II, which was a clonal lineage distinct from those found in humans. Furthermore, no *S. aureus* was isolated from handlers of those donkeys. In fact, isolates from humans were from different STs than the ones from donkeys. Three donkeys were from the same farm and all isolates presented the same resistance phenotype and genotype, which suggests a possible animal-to-animal transmission. One *S. aureus* was isolated from a donkey living in a different farm. Gharsa et al. conducted a study on *S. aureus* from donkeys destined to food consumption in Tunisia and reported a wider diversity of clones with ST133 as the main lineage (present in 44% of the isolates) which is frequently found in ruminants [13]. Little et al. studied the occurrence of *S. aureus* in diseased equines, including six donkeys, and isolated two *S. aureus* from donkeys which were ascribed to ST8-t064 and ST398-t011 [12]. *S. aureus* ST49-t208 has been widely reported as a cause of infection in European red squirrels [23,24,25] and it has also been found in wild rodents [26] and in pigs [27]. In Portugal, this particular clonal lineage has been reported in surface waters and as the dominant clone in wild night raptors [28,29]. Therefore, it seems that *S. aureus* ST49-t208 lacks host specificity, but it is associated with animals. Although studies reporting *S. aureus* in donkeys are rare, studies conducted with horses showed that the most common lineages in Europe are ST1, ST254, ST22 and ST398 [30]. Regarding the human *S. aureus* strains isolated from donkey handlers, two isolates were ascribed to ST1290, t131 and *agr* I and carried the same antimicrobial and virulence genes. Both strains were isolated from handlers from the same farm, which may suggest a possible human-to-human transmission. Furthermore, both isolates carried the IEC system genes (ascribed to type E), which indicates a human origin since the adaptation of human strains to animals required genome alterations including the loss of elements that contain the IEC [31]. *S. aureus* ST1290-t131 was first reported to be associated with community-acquired MRSA and it has also been reported among vancomycin-intermediate *S. aureus* from bloodstream infection and wild rodents from Portugal [32,33,34]. Most *S. aureus* isolates from handlers were ascribed to ST30 and *agr* III which, in turn, belonged to four different *spa*-types (t021, t338, t012 and t1642). *S. aureus* ST30 is primarily associated with human colonization and infection but it has spread to pets and farm animals [35]. *S. aureus* CC30 is associated with the carriage of the virulence genes PVL and *tst* [36,37]. In our study, four of the six ST30 *S. aureus* carried the *tst* gene but all were negative for the gene encoding PVL. Finally, two handlers’ *S. aureus* isolates belonged to ST398 (CC398) and *spa*-types t571 and t011. *S. aureus* ST398 is a lineage initially described as colonizing livestock pigs and later other farm animals, such as poultry, horses, cows and veal calves, and it has also been described as colonizing humans [38,39,40,41]. Most ST398 found among humans are associated with methicillin-susceptible *S. aureus*, whereas in animals ST398 is more often found linked to MRSA strains [42,43]. Furthermore, most ST398 *S. aureus* isolated from humans belong to *spa*-type t571 while t011 is more predominant in animals [43]. In fact, in our study, the ST398-t011 isolate was resistant to tetracycline carrying the *tet*L and *tet*M genes and according to other studies tetracycline-resistance may be a phenotypic marker of animal-associated ST398 [44]. By contrast, the ST398-t571 isolate not only lacked tetracycline resistance but it was also susceptible to all antimicrobials tested. Some of the donkey handlers tested in this study live off agriculture and it is very likely that they have more farm animals; the detection of a tetracycline-resistant ST398-t011 *S. aureus* may thus have actually resulted from an animal-to-human transmission.

MRCoNS were detected in almost half of the donkeys (42.9%) and in four (17.4%) handlers. Although studies showing the frequency of *S. aureus* in donkeys are scarce, studies of CoNS are even rarer. Moreover, the few studies conducted with donkeys focused on non-*S. aureus* staphylococci and determined only one species, mainly *S. pseudintermedius* [45,46]. Foti et al. described the bacterial flora present in the normal conjunctiva of donkeys and detected four species of CoNS species including *S. xylosus* (*n* = 22), *S. chromogenes* (*n* = 4), *S. cohnii* (*n* = 2) and *S. lentus* (*n* = 2) [20]. In the same study, CoNS were more frequent than *S. aureus*, which is in accordance with our results [20]. In a study by Gutema et al., CoNS were isolated from 9.2% of healthy donkeys and 10.6% of donkeys with respiratory problems [21]. However, in that study, the CoNS species were not identified. *S. sciuri* was the most prevalent species among donkeys. *S. sciuri* and *S. lentus* were also among the most frequently detected CoNS species in horses [47,48,49,50,51]. However, other studies report other species of CoNS as the most common in horses such as *S. capitis* and *S. equorum* [14,52]. *S. lentus* is commonly detected among farm animals [53]. Staphylococci from the *S. sciuri* group (*S. sciuri*, *S. lentus*, *S. xylosus*) are commensal bacteria of the skin and mucous membranes of different animal species and are known to have a broad host range [48]. Nevertheless, they are occasionally responsible for opportunistic infections both in animals and humans [54,55,56]. All *S. sciuri* isolates carried the *mec*A gene, including the strain isolated from one handler, as previously reported among equine staphylococcal isolates, which was not a surprise since it had been hypothesized that *S. sciuri* may be the evolutionary precursor of the *mec*A gene [4,52,57]. One handler was colonized by *S. sciuri* and one of their donkeys (VS3127) was also a carrier of *S. sciuri*. Transmission of *S. sciuri* strains between horses and their handlers have been previously documented [58]. Three handlers were colonized by *S. epidermidis*, which are one of the most common members of the healthy cutaneous microbiome both in humans and animals [59,60]. However, two *S. epidermidis* isolates were multidrug-resistant. Furthermore, although *S. epidermidis* possesses fewer virulence factors than *S. aureus*, two of the isolates carried virulence genes [61]. *eta* is an exfoliative toxin and was detected in two CoNS isolates—one *S. epidermidis* and one multidrug-resistant *S. sciuri*—and it has been previously reported in different staphylococcal species [62,63]. The *S. sciuri* isolate carrying the *eta* gene was also the only isolate showing resistance to linezolid conferred by the *optr*A gene. This gene was first found in enterococci in China, and later in *S. sciuri* isolated from pigs [64,65,66]. By contrast to *cfr*, which also confers resistance to linezolid and other antimicrobial classes, *optrA* only confers resistance to oxazolidinones [64].

## 4. Materials and Methods

### 4.1. Sample Collection and Bacterial Isolates

Oral and nasal swab samples (one swab per animal/handler) were collected from 49 Miranda donkeys (*Equus asinus*) and 23 handlers from October 2019 to January 2020 in collaboration with the Association for the Study and Protection of Donkey Cattle (Associação para o Estudo e Proteção do Gado Asinino—AEPGA). Donkeys were housed in 15 different farms. The number of samples collected in each farm are shown in Table S1. The 49 donkeys consisted of 12 males and 37 females and ranging in age from 4 months to 21 years, with a median age of 8 years (Table S1). The handlers consisted of 13 men and 10 women with a median age of 54 years. All samples were correctly identified and sent to the laboratory within a maximum of 2 days after being collected. The swabs were inserted into tubes containing brain heart infusion (BHI) broth with 6.5% of NaCl and incubated at 37 °C for 24 h. Then, 100 µL of inoculum was seeded onto Baird–Parker agar and oxacillin resistance screening agar base (ORSAB) plates for the isolation of *S. aureus* and methicillin-resistant staphylococci. Baird–Parker plates were incubated at 37 °C for 24 h whereas ORSAB plates were incubated for 24 to 48 h. One colony was recovered from each plate. The isolate species were identified by matrix-assisted laser desorption/ionisation–time of flight mass spectrometry (MALDI-TOF MS).

### 4.2. Phenotypic Antimicrobial Resistance and Susceptibility

The resistance phenotype of each of the isolates was established by the Kirby–Bauer disk diffusion method. The susceptibility of the isolates was tested against 14 antimicrobial agents according to the standards of the European Committee on Antimicrobial Susceptibility Testing (EUCAST, 2018) [67] with the exception of kanamycin, which followed the American Guidelines of the Clinical & Laboratory Standards Institute (CLSI 2017) [68] at the following concentrations per disk: penicillin 1 U, cefoxitin 30 μg, tetracycline 30 μg, linezolid 10 μg, timetroprim/sulfamethoxazole 1.25/23.75 μg, ciprofloxacin 5 μg, erythromycin 15 μg, clindamycin 5 μg, gentamicin 10 μg, tobramycin 10 μg, chloramphenicol, 30 μg, fusidic acid 10 μg, kanamycin 30 μg and mupirocin 200 µg. *S. aureus* strain ATCC 25,923 was used as quality control in the susceptibility assays.

### 4.3. Detection of Antimicrobial and Virulence Genes

According to the phenotypic resistance of each isolate, the following antimicrobial resistance genes were studied by PCR: ß-lactams (*bla*Z and *mec*A), macrolides and lincosamides (*erm*A, *erm*B, *erm*C, *erm*T, *msr* (A/B), mphC, *lnu*A, *lnu*B, *vga*A and *vga*B), tetracycline (*tet*M, *tet*K, *tet*L and *tet*O), aminoglycosides (*aac* (6′)-Ie-*aph* (2″)-Ia, *ant* (4′)-Ia, *aph* (3′)-IIIa and *str*), trimethoprim/sulfamethoxazole (*dfr*A, *dfr*G, *dfr*K and *dfr*D) and fusidic acid (*fus*A, *fus*B, *fus*C and *fus*D) [69].

The presence of *hla*, *hlb*, hld, *lukF*/*lukS*-PV, *eta*, *etb* and *tst* genes encoding for virulence factors such as hemolysins, Panton–Valentine leucocidin, exfoliatins and toxic shock syndrome toxin were investigated by PCR [69]. In addition, the presence of the IEC was also evaluated, first by investigating the presence of scn gene and then, in positive isolates, the presence of the other IEC genes (*chp*, *sak*, *sea* and *sep*) to determine the IEC group [70]. Positive and negative controls used in all experiments belonged to the strain collection of the University of Trás-os-Montes and Alto Douro.

### 4.4. Molecular Typing

All *S. aureus* isolates were typed by multilocus sequence typing (MLST) based on seven housekeeping genes (*arc*C, *aro*E, *glp*F, *gm*K, *pta*, *tpi*A and *yqi*L) as described by Enright et al. [71]. Each isolate was assigned to a ST and then to a CC according to the MLST database (http://www.mlst.net/, accessed on 14 December 2021). The isolates were also typed by *spa*-typing described by Harmsen et al. [72]. PCR products were subjected to DNA sequencing and the isolates were assigned to the specific *spa* types according to the Ridom SpaServer database (http://www.spaserver.ridom.de, accessed on 13 December 2021). Finally, *agr* typing was also performed in all *S. aureus* strains as previously described [73].

## 5. Conclusions

*S. aureus* was detected in 8% of the donkeys screened. However, colonization with different species of MRCoNS isolates with multidrug resistance were detected. *S. aureus* transmission between donkeys and their handlers appears not to have occurred since the clonal types of *S. aureus* isolates were distinct from each other. Furthermore, most *S. aureus* clones isolated from handlers were associated with human origin except for one CC398 isolate harboring resistance to tetracycline.

## Figures and Tables

**Table 1:** Genetic characterization and molecular typing of *S. aureus* strains recovered from donkeys and their handlers.

Isolate	Source	Antimicrobial Resistance		Virulence Factors		Molecular Typing		
		Phenotype	Genotype	IEC Type	Other Genes	ST (CC)	*spa*	*agr*
**VS3101**	Human	PEN, CN, TOB, KAN	*bla*Z, *aph* (3′)-IIIa	E	*hla*, *hld*, *eta*	1290 (1)	t131	I
**VS3102**	Human	PEN, CN, TOB, KAN	*bla*Z, *aph* (3′)-IIIa	E	*hla*, *hld*, *eta*	1290 (1)	t131	I
**VS3103**	Human	PEN, CN, TOB, KAN	*bla*Z, *aph* (3′)-IIIa		*hla*, *hlb*, *hld*, *tst*	30 (30)	t021	III
**VS3104**	Human	PEN, FD	*bla*Z		*hla*, *hlb*, *hld*, *tst*	30 (30)	t338	III
**VS3105**	Human	PEN, CN, KAN	*bla*Z, *aph* (3′)-IIIa		*hla*, *hlb*, *hld*, *tst*	30 (30)	t012	III
**VS3106**	Human	PEN, CN, KAN	*bla*Z, *aph* (3′)-IIIa		*hla*, *hlb*, *hld*, *tst*	30 (30)	t012	III
**VS3107**	Human	PEN, FD	*bla*Z		*hla*, *hld*	30 (30)	t1642	III
**VS3108**	Human	PEN, CN, KAN	*bla*Z, *aph* (3′)-IIIa		*hla*, *hlb*, *hld*	30 (30)	t1642	III
**VS3109**	Human	Susceptible			*hla*, *hlb*, *hld*, *eta*	398 (398)	t571	I
**VS3110**	Human	PEN, CN, TOB, KAN, TET	*bla*Z, *aph* (3′)-IIIa, *tet*L, *tet*M		*hla*, *hlb*, *hld*	398 (398)	t011	I
**VS3111**	Donkey	PEN, KAN	*bla*Z, *aph* (3′)-IIIa		*hla*, *hlb*, *hld*, *tst*	49	t208	II
**VS3112**	Donkey	CN, KAN, TET	*aph* (3′)-IIIa, *tet*K		*hla*, *hlb*, *hld*	49	t208	II
**VS3113**	Donkey	PEN, KAN	*bla*Z, *aph* (3′)-IIIa		*hla*, *hlb*, *hld*	49	t208	II
**VS3114**	Donkey	PEN, KAN	*bla*Z, *aph* (3′)-IIIa		*hla*, *hlb*, *hld*	49	t208	II

Abbreviations. PEN: penicillin; CN: gentamicin; TOB: tobramycin; KAN: kamaycin; TET: tetracycline; FD: fusidic acid; ST: sequence type; CC: clonal complex.

**Table 2:** Diversity, antimicrobial resistance and virulence of CoNS isolated from donkeys and handlers.

Isolate	Source/Species	Antimicrobial Resistance		Virulence Factors
		Phenotype	Genotype	
**VS3115**	Human/*S. epidermidis*	PEN, CN, TOB, KAN, TET, SXT	*bla*Z, *aac* (6′)-Ie-*aph*(2″)-Ia, *aph* (3′)-IIIa, *dfr*A	
**VS3116**	Human/*S. epidermidis*	PEN, CN, KAN, ERY, SXT	*mec*A, *erm*C, *aac* (6′)-Ie-*aph* (2″)-Ia, *aph* (3′)-IIIa, *dfr*A, *dfr*G	*tst*
**VS3117**	Human/*S. epidermidis*	PEN, FD	-	*eta*
**VS3118**	Human/*S. sciuri*	PEN, FOX, CD, FD	*mec*A, *lnu*A	
**VS3119**	Donkey/*S. lentus*	FD	-	
**VS3120**	Donkey/*S. lentus*	PEN, CIP, ERY, CD, TET, FD	*mec*A, *bla*Z, *lnu*A	
**VS3121**	Donkey/*S. xylosus*	TET	-	
**VS3122**	Donkey/*S. vitulinus*	PEN, TET	*mec*A	
**VS3123**	Donkey/*S. sciuri*	PEN, CD, FD	*mec*A	
**VS3124**	Donkey/*S. sciuri*	PEN, FD	*mec*A	
**VS3125**	Donkey/*S. sciuri*	PEN, FOX, TOB, KAN	*mec*A, *aph* (3′)-IIIa	
**VS3126**	Donkey/*S. sciuri*	PEN, FOX, LNZ, ERY, CN, TOB, KAN	*mec*A, *optr*A, *erm*B, *aph* (3′)-IIIa	*eta*
**VS3127**	Donkey/*S. sciuri*	PEN, FOX, CD, FD	*mec*A	
**VS3128**	Donkey/*S. sciuri*	PEN, CN, FD	*mec*A, *aph* (3′)-IIIa	
**VS3129**	Donkey/*S. sciuri*	PEN, CN, TOB, KAN, FD	*mec*A, *aph* (3′)-IIIa, *ant* (4′)-Ia, *str*	
**VS3130**	Donkey/*S. sciuri*	PEN	*mec*A	
**VS3131**	Donkey/*S. sciuri*	PEN	*mec*A	
**VS3132**	Donkey/*S. sciuri*	PEN	*mec*A	
**VS3133**	Donkey/*S. sciuri*	PEN	*mec*A	
**VS3134**	Donkey/*S. sciuri*	PEN	*mec*A	
**VS3135**	Donkey/*S. sciuri*	PEN	*mec*A	*hla*
**VS3136**	Donkey/*S. sciuri*	PEN, TET	*mec*A, *tet*K	
**VS3137**	Donkey/*S. sciuri*	PEN	*mec*A	
**VS3138**	Donkey/*S. sciuri*	PEN, KAN, TOB, TET	*mec*A, *aph* (3′)-IIIa, *ant* (4′)-Ia, *str*, *tet*M	
**VS3139**	Donkey/*S. sciuri*	PEN, CD, FD	*mec*A	

Abbreviations. PEN: penicillin; FOX: cefoxitin; LNZ: linezolid; CIP: ciprofloxacin; CN: gentamycin; TOB: tobramycin; KAN: kanamycin; ERY: erythromycin; CD: clindamycin; TET: tetracycline; FD, fusidic acid; SXT: trimethoprim-sulfamethoxazole.

## Data Availability

Not applicable.

## Supplementary Materials

The following supporting information can be downloaded at: https://www.mdpi.com/article/10.3390/antibiotics11030374/s1, Table S1: Description of donkeys and handlers’ samples, farms, sampling locations and staphylococci recovery.
