# Supplementary material for: A One Health Approach Molecular Analysis of Staphylococcus aureus Reveals Distinct Lineages in Isolates from Miranda Donkeys (Equus asinus) and Their Handlers
Source: Antibiotics (Basel). 2022 Mar 10;11(3):374. doi: 10.3390/antibiotics11030374 (PMC8944429; doi:10.3390/antibiotics11030374)
Supplement: Supplementary file 1 [file antibiotics-11-00374-s001.zip › antibiotics-1600153-supplementary.pdf]

Article

One health genomic surveillance of *Staphylococcus aureus* reveals distinct lineages in isolates from Miranda donkeys (*Equus asinus*) and their handlers

Vanessa Silva [1,2,3,4], Cláudia Alfarela [1], Manuela Caniça [5,6], Vera Manageiro [5,6], Miguel Nóvoa [7], Belen Leiva [7], Maria Kress [7], José Luís Capelo [8,9], Patrícia Poeta [1,4,10,11,*,†] and Gilberto Igrejas [2,3,4,†]

1 Microbiology and Antibiotic Resistance Team (MicroART), Department of Veterinary Sciences, University of Trás-os-Montes and Alto Douro (UTAD), 5000-801 Vila Real, Portugal; vanessasilva@utad.pt (V.S.); claudiavpa@hotmail.com (C.A.)
2 Department of Genetics and Biotechnology, University of Trás-os-Montes and Alto Douro, 5000-801 Vila Real, Portugal; gigrejas@utad.pt
3 Functional Genomics and Proteomics Unit, University of Trás-os-Montes and Alto Douro (UTAD), 5000-801 Vila Real, Portugal
4 Associated Laboratory for Green Chemistry (LAQV-REQUIMTE), University NOVA of Lisboa, Lisboa, 2825-466 Caparica, Portugal
5 National Reference Laboratory of Antibiotic Resistances and Healthcare Associated Infections (NRL-AMR/HAI), Department of Infectious Diseases, National Institute of Health Dr Ricardo Jorge, Av. Padre Cruz, 1649-016 Lisbon, Portugal; manuela.canica@insa.min-saude.pt (M.C.); vera.manageiro@insa.min-saude.pt (V.M.)
6 Centre for the Studies of Animal Science, Institute of Agrarian and Agri-Food Sciences and Technologies, Oporto University, 4051-401 Oporto, Portugal
7 AEPGA, Association for the Study and Protection of Asinines, Atenor, 5225-011 Miranda do Douro, Portugal; miguelnovoa@aepga.pt (M.N.); belenleiva.aepga@gmail.com (B.L.); mariakress.aepga@gmail.com (M.K.)
8 BIOSCOPE Group, LAQV@REQUIMTE, Chemistry Department, Faculty of Science and Technology, NOVA University of Lisbon, 2825-466 Almada, Portugal; jlcm@fct.unl.pt
9 Proteomass Scientific Society, 2825-466 Costa de Caparica, Portugal
10 CECAV – Veterinary and Animal Research Centre, University of Trás-os-Montes and Alto Douro (UTAD), 5000-801 Vila Real, Portugal
11 Associate Laboratory for Animal and Veterinary Sciences (AL4AnimalS), Portugal
* Correspondence: ppoeta@utad.pt
† These authors contributed equally to this work

Table S1. Description of donkeys and handlers' samples, farms, sampling locations and staphylococci recovery.

Sample	Donkey/ Handler	Age (years)	Gender	Farm	Location	Isolate
B1	Donkey	3	M	1	Atenor	**VS3136**
B2	Donkey	16	F	1	Atenor	
B3	Donkey	1.5	M	1	Atenor	**VS3129**
B4	Donkey	14	M	1	Atenor	**VS3137**
B5	Donkey	2	F	1	Atenor	**VS3132**
B6	Donkey	4	F	1	Atenor	**VS3123**
B7	Donkey	13	M	1	Atenor	**VS3124**
B8	Donkey	14	F	2	Atenor	**VS3125**
B9	Donkey	0.3	M	1	Atenor	**VS3135**
B10	Donkey	8	F	1	Atenor	**VS3121**
B11	Donkey	5	M	1	Atenor	
B12	Donkey	15	F	1	Atenor	**VS3133**
B13	Donkey	10	M	1	Atenor	**VS3119**
B14	Donkey	9	F	2	Atenor	
B15	Donkey	8	F	2	Atenor	**VS3122**
B16	Donkey	8	F	1	Atenor	**VS3139**

B17	Handler	23	M	2	Atenor	
B18	Handler	23	M	1	Atenor	**VS3101**
B19	Handler	30	F	1	Atenor	**VS3102**
B20	Handler	30	F	1	Atenor	**VS3115**
B21	Handler	35	F	1	Atenor	
B22	Handler	28	M	1	Atenor	**VS3103**
B23	Handler	48	F	2	Atenor	**VS3104**
B24	Donkey	-	F	3	Malhadas	
B25	Donkey	2	F	3	Malhadas	**VS3120**
B26	Donkey	13	F	3	Malhadas	
B27	Donkey	18	F	3	Malhadas	
B28	Donkey	20	F	3	Malhadas	
B29	Donkey	-	M	5	Vila Chã	**VS3138**
B30	Donkey	-	F	6	Pena Branca	**VS3134**
B31	Donkey	-	F	6	Pena Branca	**VS3126**
B32	Donkey	-	F	6	Pena Branca	
B33	Donkey	-	F	7	Paradela	
B34	Donkey	-	F	7	Paradela	
B35	Donkey	-	F	7	Paradela	
B36	Handler	71	F	3	Malhadas	**VS3116**
B37	Handler	64	F	3	Malhadas	
B38	Handler	66	F	3	Malhadas	
B39	Handler	79	M	5	Vila Chã	**VS3109**
B40	Handler	74	M	3	Malhadas	
B41	Handler	70	F	7	Paradela	**VS3110**
B42	Handler	72	M	7	Paradela	
B43	Donkey	7	F	4	Malhadas	**VS3131**
B44	Donkey	21	F	4	Malhadas	
B45	Donkey	3	F	8	Palacar	
B46	Donkey	4	M	4	-	**VS3127**
B47	Donkey	9	F	4	Malhadas	**VS3128**
B48	Handler	71	F	8	Palancar	**VS3118**
B49	Handler	61	F	4	Malhadas	
B50	Handler	34	M	10	Bemposta	
B51	Handler	70	M	11	Mogadouro	
B52	Handler	68	M	15	Uva	**VS3117**
B53	Handler	56	M	12	Sendim	**VS3105**
B54	Handler	42	M	9	Sendim	**VS3106**
B55	Handler	69	M	14	Uva	**VS3107**
B56	Handler	65	M	13	Vila Chã da Ribeira	**VS3108**
B57	Donkey	8	F	9	Sendim	
B58	Donkey	2.5	F	14	Uva	
B59	Donkey	11	F	14	Uva	
B60	Donkey	5	F	15	Uva	
B61	Donkey	6	M	9	Sendim	
B62	Donkey	3	F	15	Uva	
B63	Donkey	4.5	F	9	Sendim	
B64	Donkey	4.5	F	13	Vila Chã da Ribeira	
B65	Donkey	8	F	11	Mogadouro	
B66	Donkey	7	F	10	Bemposta	**VS3111**
B67	Donkey	5	F	11	Mogadouro	**VS3112**

B68	Donkey	8	F	10	Bemposta	**VS3113**
B69	Donkey	3.5	M	10	Bemposta	**VS3114**
B70	Donkey	9	F	15	Uva	**VS3130**
B71	Donkey	6	F	12	Sendim	
B72	Donkey	4	M	12	Sendim	
